# Inhibition of YAP Sensitizes the Selumetinib Treatment for Neurofibromatosis Type 1 Related Plexiform Neurofibroma

**DOI:** 10.7150/ijms.78386

**Published:** 2023-01-01

**Authors:** Zhuowei Tian, Yuanhe You, Meng Xiao, Jialiang Liu, Guisong Xu, Chunyue Ma, Zhong Du, Yanan Wang

**Affiliations:** 1 Department of Oral Maxillofacial-Head and Neck Oncology, Shanghai Ninth People's Hospital, Shanghai Jiao Tong University School of Medicine; College of Stomatology, Shanghai Jiao Tong University; National Center for Stomatology; National Clinical Research Center for Oral Diseases; Shanghai Key Laboratory of Stomatology, Shanghai, China.; 2 Department of Oral Maxillofacial-Head and Neck Oncology, Fengcheng Hospital, Shanghai, China.; 3 Shanghai Stomatological Hospital, Fudan University, Shanghai 200011, China.

**Keywords:** Neurofibromatosis type 1, Plexiform neurofibroma, Selumetinib, YAP, Targeted therapy

## Abstract

**Background:** Targeted therapy of Neurofibromatosis type 1 (NF1) related plexiform neurofibroma (pNF) aiming at MEK molecule has not demonstrated a convincing result for complete disease inhibition, probably due to other signal pathways crosstalk. Our previous study revealed an increased nuclear translocation of YAP molecule in NF1 related pNF. Herein, we decided to further investigate the therapeutic relations of YAP interference during the MEK treatment against NF1 related pNF.

**Methods:** By means of selumetinib (MEK-inhibitor), RNA-sequencing was firstly performed to identify the changes of signal pathways in pNF Schwann cells, which was probably related to YAP regulation. Nuclear-cytoplasmic fractionation and western blotting were performed to show the intracellular YAP changes under selumetinib treatment. Thirdly, a series of *in vitro* assays were performed including flow cytometry, CCK-8, and colony/sphere formation under dual treatment of selumetinib and verteporfin (YAP-inhibitor). In addition, Chou-Talalay method was adopted to evaluate the synergistic inhibiting effects of such drug combination. Xenograft study was also used to detect the combining effects *in vivo*.

**Results:** RNA-sequencing revealed that selumetinib treatment might be associated with the undesirable activation of Hippo pathway in NF1 related pNF tumor cells, which might reduce its pharmaceutic effects. Next, nuclear-cytoplasmic fractionation and further studies demonstrated that selumetinib could promote the nuclear translocation and transcriptional activation of YAP *in vitro*, which might cause the aforementioned resistance to selumetinib treatment. Additionally, when combined treatments were performed based on verteporfin and selumetinib, synergistic effects were observed on cytotoxicity of NF1 related pNF tumor cells *in vitro* and* in vivo* xenograft models.

**Conclusion:** YAP inhibition can effectively sensitize NF1 related pNF tumor cells to selumetinib. Dual targeting of YAP and MEK might be a promising therapeutic strategy for treating NF1 related pNF.

## Introduction

Neurofibromatosis type 1 (NF1) is an inherent tumor predisposition syndrome, with an incidence frequency of 1/3000 worldwide 1, 2. Approximate 90% of the patients ultimately develop cutaneous and/or plexiform neurofibromas (pNFs) 3. pNFs are benign perineural Schwann cell tumors 4 and can cause severe substantial complications. Unlike cutaneous neurofibromas, it has a lifetime risk [8-13%] to malignant transformation 5. pNF is usually considered as the greatest burden for NF1 patients, causing severe deformity and dysfunction, yet with no effective treatment strategy so far. Aggressive surgical removal of neurofibroma is usually not etiotropic, and tumor regrowth can be found after inappropriate surgical resection 6, 7. In an attempt to cure the disease, attention has been recently shifted towards targeted or biologic therapy. Within all the changed signals in such disease, the overactivation of the Ras/MAPK pathway, which caused by* NF1* mutation is widely acknowledged for its role in tumorigenesis. Therefore, etiologically speaking, targeting the Ras pathway with MAPK kinase (MEK) inhibition seems to be a rational treatment strategy 8. Based on this hypothesis, selumetinib, which inhibits MEK molecule, was firstly approved for treating young patients (≥2 years old) with symptomatic and inoperable pNF by the FDA in 2020 (NCT01362803) 9, 10. The results indicated about 68% of patients shows approximately 20% tumor shrinkage, indicating that additional work is necessary to dramatically reduce tumor burden 11.

Apart from MEK, other molecular factors have been also found to participate in regulating or curbing the neurofibroma formation, such as RUNX 12, STAT3 13 and YAP 14. Hence, it seems to be reasonable to explore new drug targets for NF1 patients, especially for those with tolerance/insensitivity to MEK inhibitor 15. YAP is a core effector of the Hippo signaling pathway, which plays an essential role in cancer, fibrosis and other diseases 16. Thus, we came up with the idea of finding the potential role of YAP in patients with MEK inhibition. Dual targeting of YAP and MEK may be a more effective combination strategy for NF1 patients with pNFs.

## Materials and Methods

### Cell culture

The human NF1 related neurofibroma Schwann cell lines (ipNF95.11bC and ipNF95.6) were obtained from American Type Culture Collection. The mouse Schwann cell line (SW10) was purchased from the Cell Bank of Chinese Academy of Sciences (Shanghai, China). All the Schwann cells were cultured by Dulbecco's modified Eagle medium (DMEM) (Gibco, USA) that contains 10% FBS and 1% penicillin/streptomycin in a fully humidified atmosphere (37°C, 5%CO_2_).

### RNA-sequencing

RNA transcriptome profiling was carried out for the ipNF95.6 Schwann cells after selumetinib treatment by Illumina (USA) high-throughput RNA sequencing. RNA was collected by RNeasy Plus Mini Kit according to manufacturer (QIAGEN, Cat. 74136, Germany). The transcript quantification and normalization were implemented using RSEM software package. Genes with significant down- or up-regulation (Q-value≤0.05 and fold change >1.5) were identified as differentially expressed genes (DEGs). The DEGs were filtered to functional classification using GO, KEGG, and GSEA analysis.

### RNA interference

Small hairpin RNA (shRNA) sequences targeting* Nf1*, plasmids, and lentiviruses were synthesized by Genomeditech Inc. (China). The efficiency of the shRNA was validated by using Western blot and qRT-PCR.

siRNA targeting YAP was produced by Genomeditech Inc. (China) and the tumor cells were transfected by using Lipofectamine® 3000 Transfection Kit (L3000015, Invitrogen, USA). A scrambled siRNA was adopted as negative control. The target siRNA sequence used were: YAP (human) (5'-AUGACAACCAAUAGUUCAG-3'); Yap (mouse) (5'-ACUUGGAGGCGCUCUUCAAUG-3'). The efficiency of siYAP was identified by Western blot and qRT-PCR.

### Western blot (WB) assay

Whole protein was isolated by using whole-cell lysis buffer in the presence of a protein phosphatase inhibitor cocktail (Pierce, USA). For detecting the YAP nuclear translocation, Nuclear-cytoplasmic fractionation was conducted using the NE-PER Nuclear and Cytoplasmic Extraction Reagents kit (Thermo Fisher Scientific, USA) according to the manufacturer's protocol, which enable stepwise separation and preparation of cytoplasmic and nuclear extracts from Schwann cell lineage. After the samples were electrophoresed through 8-12% polyacrylamide gels, proteins were transferred onto a PVDF membranes. The membranes were incubated with primary antibodies. Antibodies include (Cell Signaling Technology, USA, unless otherwise indicated): NF1 (D7R7D) (1:1,000, #14623) YAP (D8H1X) (1:1,000, #14074), p-YAP (S127) (D9W2I) (1:500, #13008S), β-Actin (13E5) (1:1,000, #4970), Cleaved Caspase-3 (Asp175) (1:1,000, #9664), GAPDH (D4C6R) (1:1,000, #97166) and Histone H3 (1:1,000, affinity # BF9211). After incubated with peroxidase-linked secondary antibodies (GK500705, Gene Tech, China), the membranes were washed and visualized using Immobilon Western HRP Chemiluminescence Substrate (Millipore, USA).

### Immunofluorescence assay

For immunofluorescence analysis, cells were fixed and blocked. Then, the cells were incubated with primary antibodies YAP (D8H1X) (1:1,00, #14074) overnight at 4 °C. Secondary anti-rabbit antibodies labeled either with Alexa Fluor® 594 dye (red) from Cell Signaling Technology (USA). DAPI (CST, USA) was used at 1:500 dilutions. Images of the cells were taken with a BD Beckman cytometer (BD Biosciences, USA).

### Real-time quantitative PCR analysis

Total RNA was isolated using TRIZOL Reagent (Invitrogen, USA) and subsequently reverse transcribed into cDNA using PrimerScript RT reagent Kit (Takara, Japan) according to the manufacturer's guideline. The cDNA was subjected to qRT-PCR detection using an ABI StepOne real-time PCR system (Life Technologies, USA). The PCR primers used in these studies are described in Table S1. The relative expression was calculated using the 2^-ΔΔCT^ method. The primers used in our study were shown in Table S1.

### Cell Counting Kit-8 (CCK-8) assay

Cytotoxicity of the compounds was measured by CCK-8 kit (New Cell&Molecular Biotech, China). Schwann cells were seeded in 96-well plates at 3000 cells/well and incubated overnight for attachment. MEK1/2 inhibitor selumetinib (AZD6244) and YAP inhibitor verteporfin were obtained from Selleck Chemicals (China). Cells were treated with selumetinib (Range, 0-200 μM) and/or verteporfin (Range, 0-10 μM) for 72h. This medium was replaced with 10% CCK-8 and incubated at 37 °C for 2 hours. The absorbance was measured at 450 nm. The IC50 values were calculated via Graphpad prism 8.0 software (USA).

### Assessment of Synergistic Effects

Combination indices (CI) of drug combinations were evaluated via the Chou-Talalay method using CompuSyn software (ComboSyn Inc., Paramus, USA) 17, and CI values CI<1.10 was identified as synergism; CI values >1.1 were considered as antagonism 18.

### Apoptosis assay

Apoptotic levels of involved cells were detected after drugs treatment by flow cytometry using an Annexin V-APC/PI Apoptosis Detection Kit (BD, USA). Cells were collected after different drug treatments, then the assays were carried out according to the manufacturer's protocol. Samples were processed immediately using a FACSCalibur flow cytometer (BD, USA). Data was further analyzed by FlowJo 10.4. 20,000 cells were recorded and analyzed in all conditions.

### Colony formation assay

ipNF95.11bC, ipNF95.6 and shNf1-SW10 cells were seeded in 6-well plates (1000 cells/well) and incubated for 2 weeks. The colonies formed were washed with PBS and fixed with 4% paraformaldehyde, subsequently stained with 1% crystal violet for 20 min at room temperature. All visible cell colonies were imaged and counted.

### Sphere formation assay

Cells were seeded at 1000 cells/well of at 6 wells low adhesion plate (Corning Inc., USA). Micro spheres were cultured in DMEM/F12 medium added with vitamin B27, heparin (4 mg/mL) and FGF (20 ng/mL). Samples were subsequently treated with selumetinib and verteporfin, alone or combination, and equal DMEM was used as control. All visible spheres were imaged and counted after 2 weeks of continuous medium exposure.

### *In vivo* xenograft studies

All animal experimental procedures were approved by the Animal Welfare Committee of the Ninth People's Hospital, Shanghai Jiao Tong University School of Medicine (China). shNf1-SW10 cells (2×10^6^ cells per injection) were subcutaneously injected into 4-week-old BALB/c nude mice model (n=24). When the maximum diameter of tumors reached 1cm, the mice were randomly divided into four groups (i) vehicle, (ii) selumetinib (50 mg/kg) orally every other day, (iii) verteporfin (20 mg/kg) intraperitoneally every other day or (iv) the combination of selumetinib and verteporfin, respectively. All the mice were sacrificed and the tumors were excised and then fixed with 4% Paraformaldehyde Fix Solution for 24 hours for further analysis. The ellipsoid volume formulas (π/6 * L * W * H) were used for estimating tumor mass.

### Statistical analysis

Statistical analysis was performed using GraphPad prism 8.0 software (USA). A Student's t-test was used to analyze differences between two groups and one-way ANOVA was adopted to analyze more than two groups. A *p* value < 0.05 was considered as statistically significant.

## Results

### Increased transcriptional activation of Hippo signaling pathway in human neurofibroma cells under selumetinib treatment

**Figure 1:**
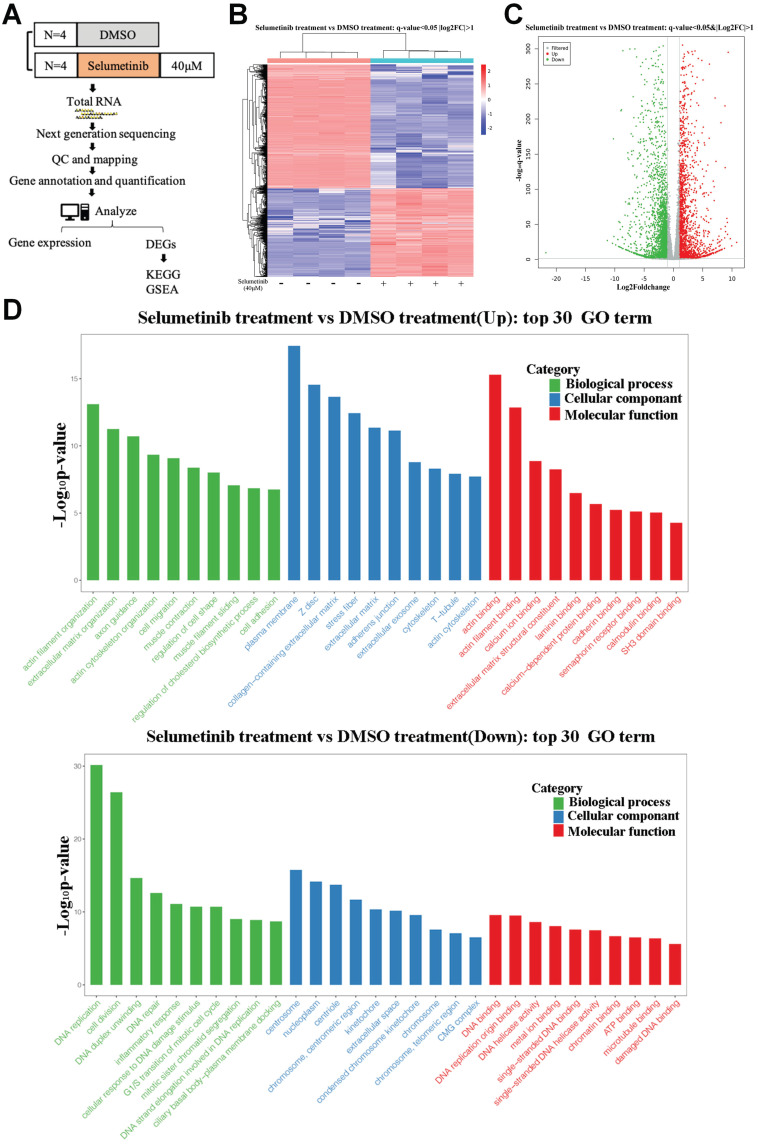
** mRNA expression profile was analyzed after selumetinib treatment. (A)** Workflow of RNA-sequencing experiment. Total RNA of human neurofibroma Schwann cell (ipNF95.6) was collected after selumetinib or DMSO treatment for 72h. **(B)** The heatmap showed hierarchical clustering of differentially expressed genes between selumetinib treatment and control group. **(C)** Volcanic plots for the mRNA sequencing of ipNF95.6 cells treated by selumetinib or DMSO. **(D)** GO analysis for the up-regulated (Up) or down-regulated (Down) differential expression genes.

**Figure 2:**
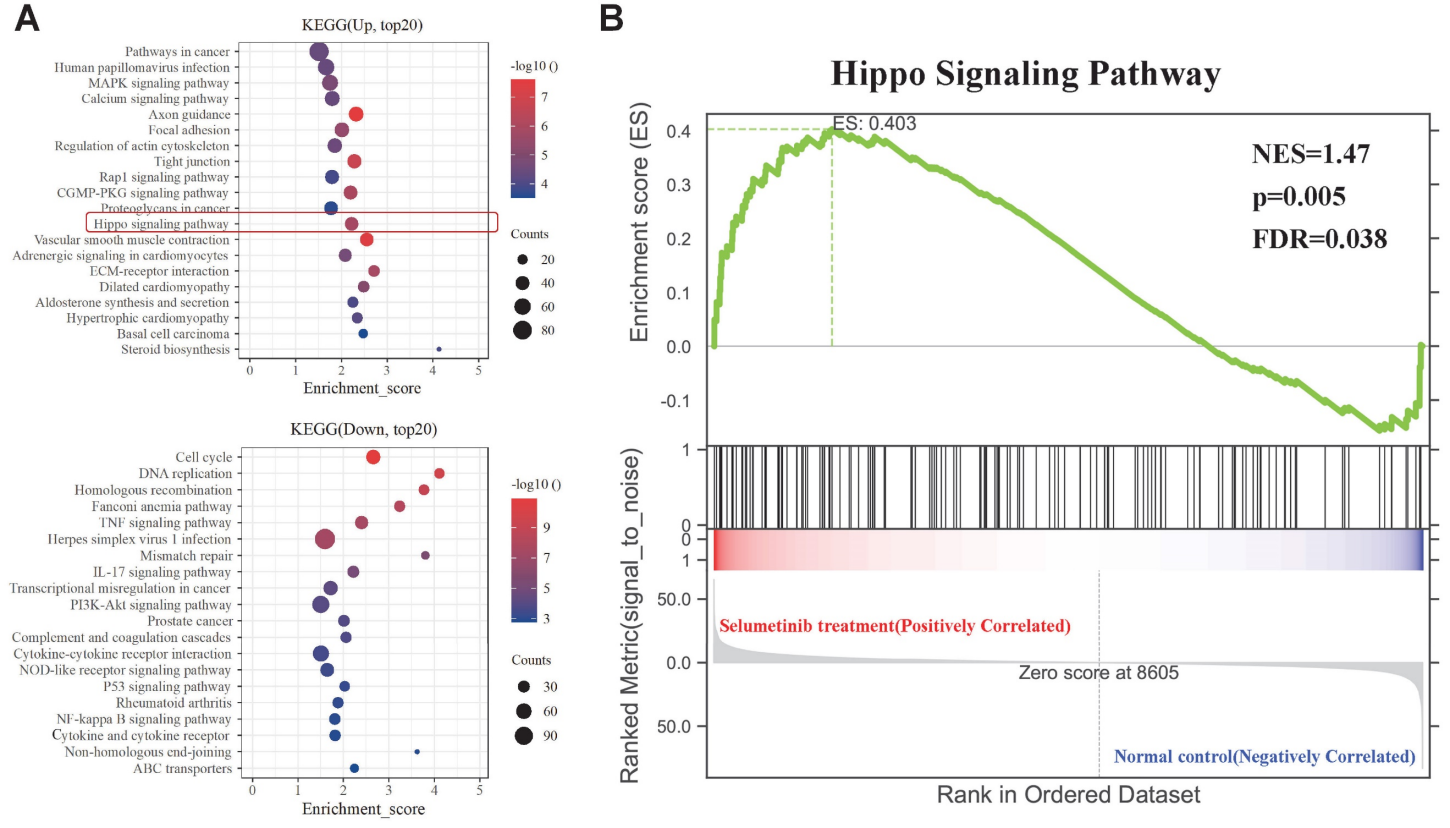
** KEGG and GSEA analysis of the DEGs. (A)** KEGG analysis for the differential expression genes; Up: Up-regulated; Down: down-regulated. **(B)** GSEA plots showed normalized enrichment score (NES) of Hippo signaling pathway signature in differentially expressed genes.

After treatment with selumetinib, human neurofibroma cells were harvested for further RNA sequencing analysis to assess the potential molecule of selumetinib resistance (Figure 1A). As shown in Figure 1B and 1C, a total of 4888 genes were filtered and identified as differentially expressed genes (DEGs, Q value <0.05 and Fold change >2). GO analysis for the DEGs indicated that selumetinib could affect the cell division, cell cycle, cell migration, matrix organization of neurofibroma cells (Figure 1D). KEGG enrichment analysis for DEGs revealed the dysregulated signaling pathways (Figure 2A). Among these pathways, Hippo pathway, which plays an important role in the tumorigenesis of neurofibroma, was proved to involve in RTKs or RAF drug resistance. GSEA analysis further confirmed that selumetinib positively associated with Hippo transcriptional activation level (Figure 2B). The above information indicated that Hippo could be activated by selumetinib treatment and may be a potential molecule in selumetinib resistance.

### Selumetinib promoted nuclear accumulation and activation of YAP in NF1-neurofibroma Schwann cells

**Figure 3:**
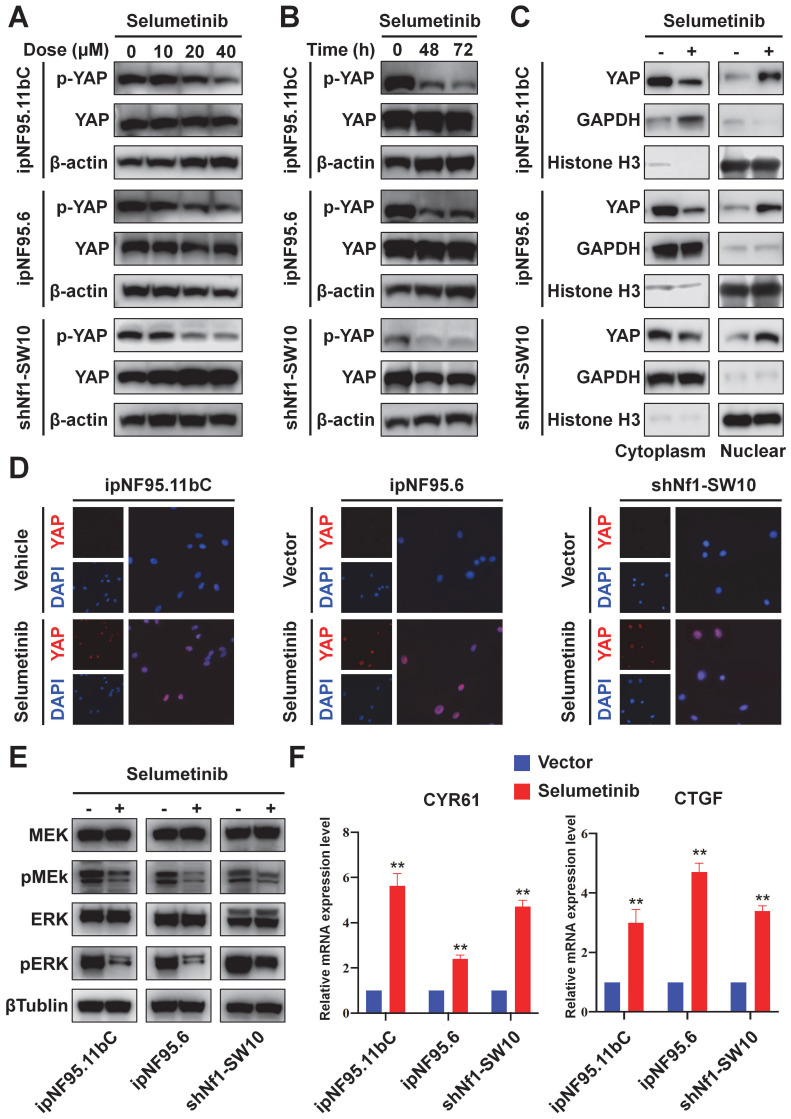
**Selumetinib treatment promoted the nuclear translocation and activation of YAP in neurofibroma Schwann cells. (A)** Human neurofibroma Schwann cell (ipNF95.11bC and ipNF95.6) and shNf1-SW10 cells were intervened with selumetinib with indicated dose (from 0 to 40 μM) for 48 hours. The cells were harvested to evaluated YAP functional status through Western blotting. **(B)** The YAP protein status of Human neurofibroma Schwann cell (ipNF95.11bC and ipNF95.6) and shNf1-SW10 cells were assessed by Western blotting after treated with selumetinib (40 μM) for indicated time (0, 48 and 72 hours). **(C)** Cytoplasmic/nuclear proteins were separated according manufacture's guideline. Increased nuclear accumulation of YAP in ipNF95.11bC, ipNF95.6 and shNf1-SW10 cells were detected by Western blotting. **(D)** Immunofluorescence identified the increased nuclear accumulation of YAP in ipNF95.11bC, ipNF95.6 and shNf1-SW10 cells after treating with selumetinib. **(E)** MEK and ERK phosphorylation level changed in ipNF95.11bC, ipNF95.6 and shNf1-SW10 cells after treating with selumetinib. **(F)** mRNA expression of YAP target genes, CYR61and CTGF, analyzed by qRT-PCR in ipNF95.11bC, ipNF95.6 and shNf1-SW10 cells after treating with selumetinib. The results were presented as mean ± SEM (n = 3) of three-independent assays. *p < 0.05, **p < 0.01.

YAP phosphorylation levels were detected to further evaluate the functional status of Hippo pathway. Human neurofibroma Schwann cells (ipNF95.11bC and ipNF95.6) and murine neurofibroma cells model (shNf1-SW10, Figure S1A&B) were harvested after selumetinib exposure. A significant decrease in YAP phosphorylation was found after selumetinib treatment by concentration-dependent manner and time-dependent manner (Figure 3A&B). To determine the localization of YAP, we managed to separate cytoplasmic and nuclear proteins and observed that selumetinib treatment resulted in the nuclear accumulation of YAP in human neurofibroma cells and shNf1 SW10 cells (Figure 3C). Immunofluorescence were also performed to show the increased accumulation of YAP in Schwann cells' nuclear after treating with selumetinib (Figure 3D). We verified that MAPK pathway was down regulated by selumetinib in human neurofibroma cells and shNf1 SW10 cells by MEK and ERK phosphorylation level using Western Blotting (Figure 3E). While, our results demonstrated the activation of YAP by measuring the mRNA expression levels of canonical YAP downstream genes, CYR61 and CTGF (Figure 3F). The above information indicated that Hippo-YAP was activated under selumetinib treatment in NF1-neurofibroma Schwann cells.

### Genetic inhibition of YAP sensitized NF1-neurofibroma Schwann cells to selumetinib

**Figure 4:**
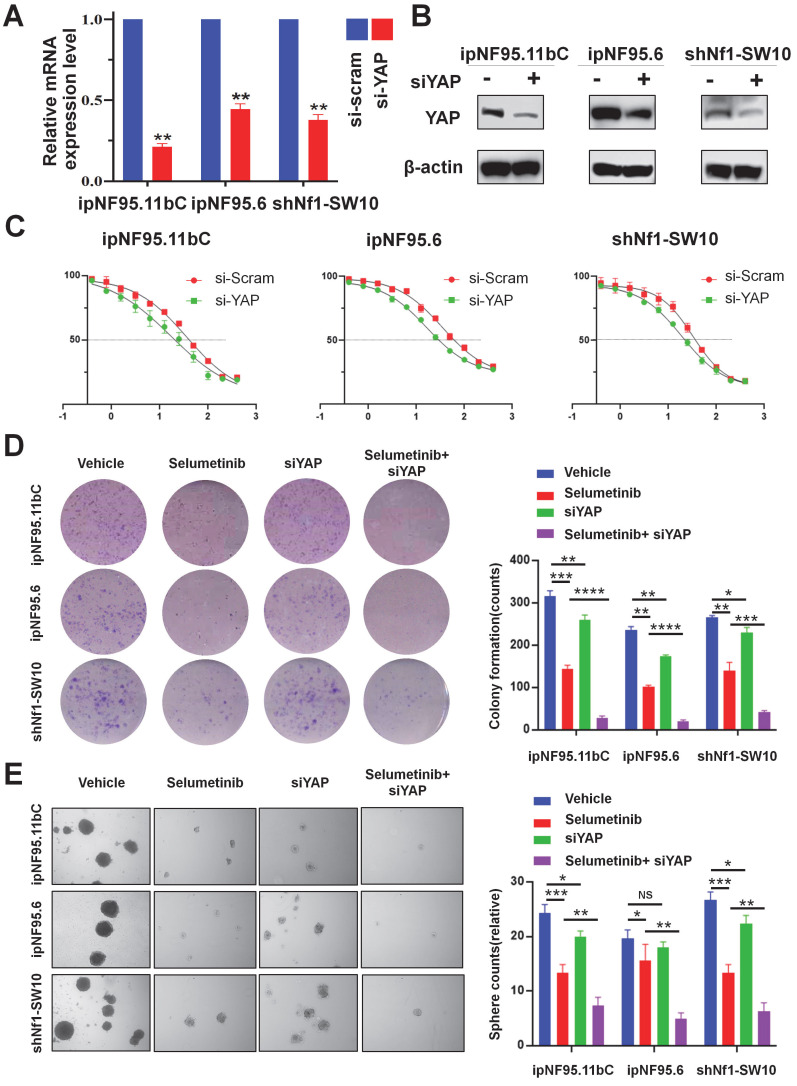
** Genetic inhibition of YAP sensitized selumetinib treatment in pNF Schwann cells. (A-B)** Transfection efficiency was detected using qRT-PCR and Western Blot after YAP-specific siRNA transfection for 72h in ipNF95.11bC, ipNF95.6 and shNf1-SW10 cells. **(C)** IC50 curves for 72h of selumetinib in neurofibroma Schwann cells (ipNF95.11bC, ipNF95.6 and shNf1-SW10 cells) transfecting with siRNA. IC50 were calculated based on a nonlinear regression analysis. **(D-E)** Proliferation ability of siYAP transfecting neurofibroma Schwann cells (ipNF95.11bC, ipNF95.6 and shNf1-SW10 cells) were detected by performing colony formation and sphere formation assay under selumetinib exposure. *p < 0.05, **p < 0.01, ***p < 0.005, ****p < 0.001.

To further confirm the impact of YAP activation in selumetinib treatment process, we inhibited YAP expression using siRNA targeting YAP. Three different siRNA sequences were used for ipNF95.11bC, ipNF95.6 and shNf1-SW10 cells. The transfection efficiency was detected by using Western blot after transfection for 48h (Figure S1C). And the most effective siRNA sequence (siYAP2 for ipNF95.11bC and ipNF95.6 cells; siYap3 for shNf1-SW10 cells) was chosen for our subsequent studies. The expression of YAP was verified at mRNA and protein level again (Figure. 4A&B). We found that the response of neurofibroma cells to selumetinib was directly related to the expression level of YAP (Figure 4C). YAP inhibition effectively sensitized both types of Schwann cells to selumetinib. The IC50 for 72 h value was shifted from 36.47 μM to 17.07 μM in ipNF95.11bC, from 34.62 μM to 16.43 μM in ipNF95.6 cells, and from 32.52 μM to 19.33 μM in shNf1-SW10 cells. We also observed that siYAP in combination with selumetinib could significantly inhibit the colony formation ability of tumor cells (Figure 4D). Moreover, the sphere numbers of pNF Schwann cells transfected with siYAP were significantly less than those of the vector control after selumetinib treatment (Figure 4E). The sphere size of pNF Schwann cells transfected with siYAP were significantly smaller than those of the vector control after selumetinib treatment (Figure S2A). Taken together, the above results indicated that YAP might exert a role in selumetinib resistance in neurofibroma cells.

### YAP inhibitor enhanced selumetinib cytotoxicity in neurofibroma cells

**Figure 5:**
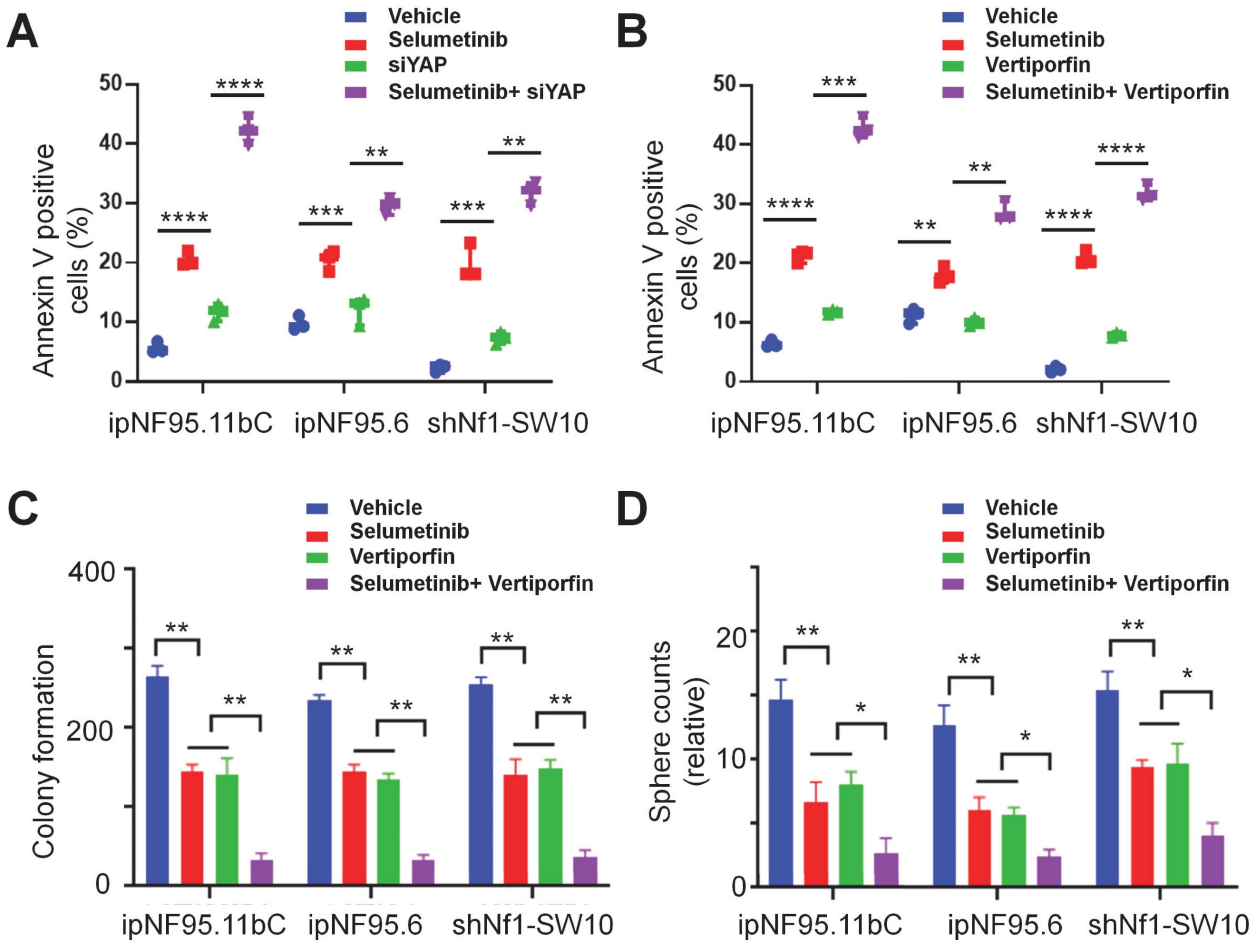
** YAP inhibitor enhanced selumetinib cytotoxicity in neurofibroma Schwann cells. (A-B)** Flow cytometry were performed to assess the cytotoxicity of selumetinib. A, Neurofibroma Schwann cells (ipNF95.11bC, ipNF95.6 and shNf1-SW10 cells) transfecting with YAP-specific siRNA were treated with or without selumetinib for 48h. B, Neurofibroma Schwann cells were treated with selumetinib, verteporfin or combination for 48 h. The results were presented as Mean ± SEM (n=3). **p < 0.01, ***p < 0.005, ****p < 0.001. **(C-D)** Colony formation and Sphere fromation numbers of neurofibroma schwann cells (ipNF95.11bC, ipNF95.6 and shNf1-SW10 cells) were detected after treating with selumetinib, verteporfin or combination. *p < 0.05, **p < 0.01.

We also assessed the apoptotic levels of neurofibroma cells by performing flow cytometry assays. Our results indicated that YAP knockdown or treatment with verteporfin (YAP inhibitor) significantly enhanced selumetinib induced apoptosis in neurofibroma cells (Figure 5A&B). Moreover, increased cleaved caspase-3 activity also indicated that YAP knockdown could contribute to selumetinib induced apoptosis (Figure S3). In addition, we observed that the differential combination of verteporfin and selumetinib resulted in a significant reduction in colony formation (Figure 5C) and sphere formation (Figure 5D&S2B).

### Combined inhibition of YAP and MEK had a synergistic impact on NF1-neurofibroma cells *in vitro*

**Figure 6:**
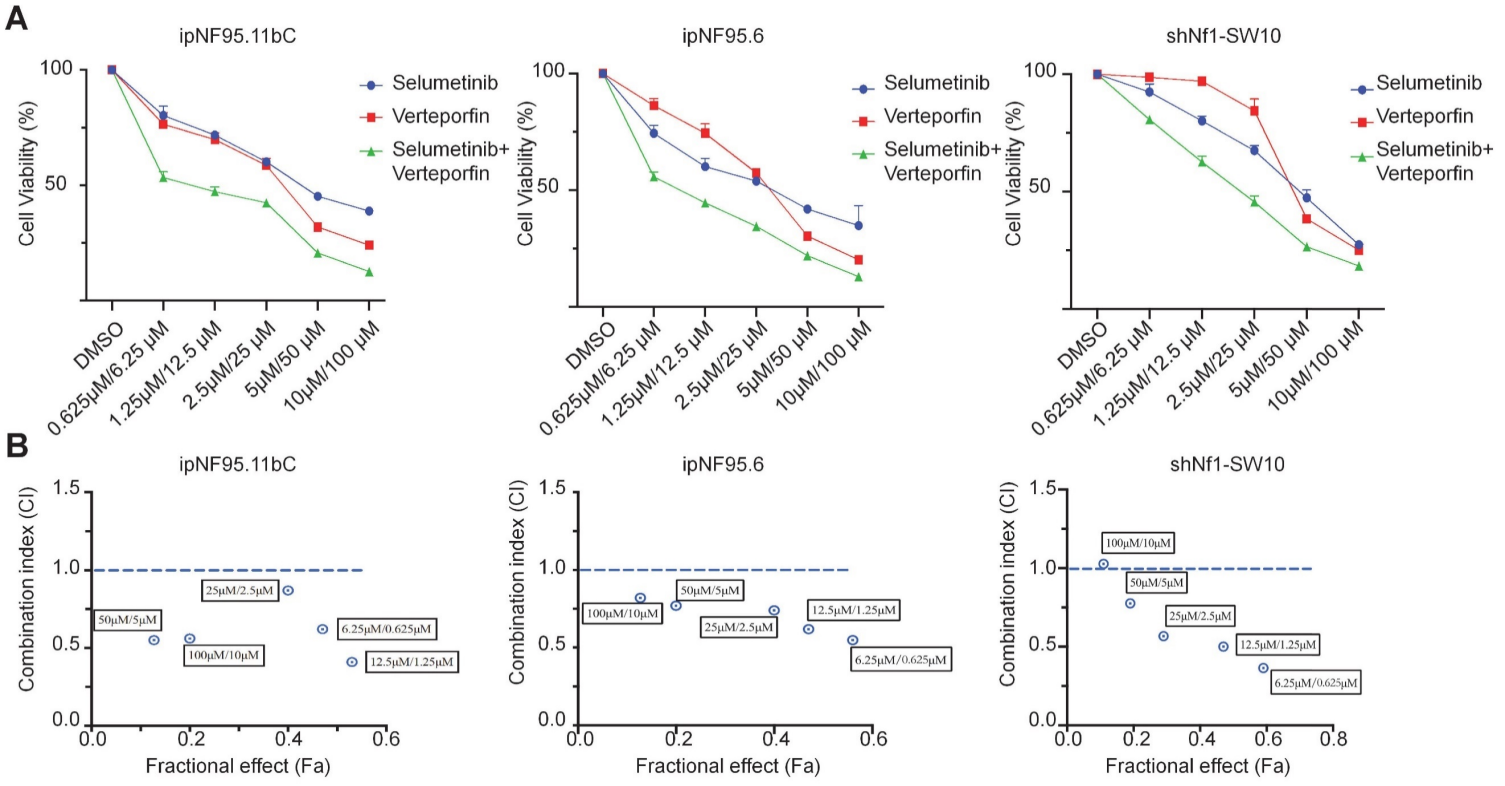
** A synergistic effect was observed in dual treatment with selumetinib and verteporfin. (A)** Cell viabilities were measured in ipNF95.11bC, ipNF95.6 and shNf1-SW10 cells using a CCK-8 assay after treating with different combinations of selumetinib and verteporfin for 72h. (selumetinib concentration range: 0-100μM; verteporfin concentration range: 0-10μM). **(B)** Combination indices (CI) were calculated via the Chou-Talalay method (Fa, Fractional effect; synergism CI<1.10; antagonistic CI>1.10).

To further elucidate the possible synergism of the dual targets of YAP and MEK, we treated the neurofibroma cells with a combination of verteporfin and selumetinib. Neurofibroma cells were exposed to either selumetinib, verteporfin or a combination *in vitro*. Cytotoxicity was detected by CCK-8 assay after 72h of incubation. Combined therapy showed a dose-dependent reduction in cellular viability (Figure 6A). Furthermore, we applied the Chou-Talalay method via CompuSyn software to calculate the combination indices (CI). Our results revealed that synergistic effects of dual targeting of YAP and MEK with the Chou and Talalay methods (Figure 6B).

### Dual targeting of YAP and MEK inhibited neurofibroma growth *in vivo*

**Figure 7:**
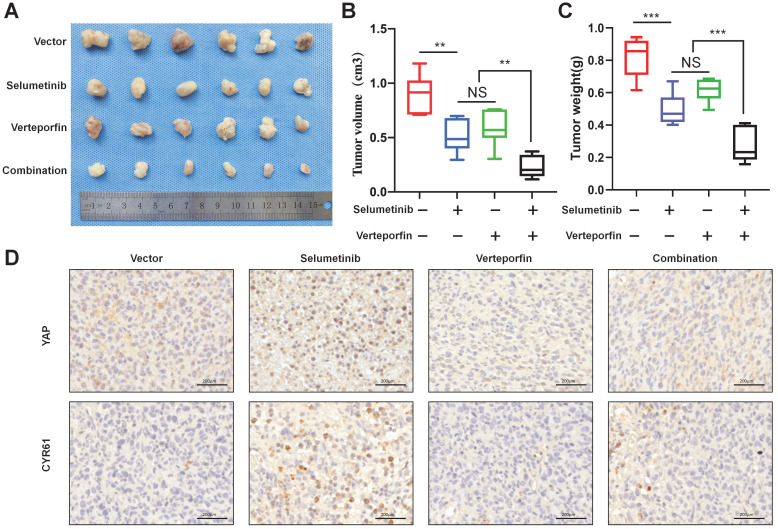
** Selumetinib in combination with verteporfin inhibited tumor growth in a shNf1-SW10 cells xenotransplantation mouse model. (A)** Treatment with selumetinib, verteporfin alone or combination reduced the tumor size in a mouse model generated by subcutaneous injection of shNf1-SW10 cells. **(B)** Quantification of tumor size among the different groups. *p < 0.05, **p < 0.01. **(C)** Quantification of tumor weight among the different groups. ***p < 0.005. **(D)** YAP status and CYR61 expression level in xenograft mass under treatment of selumetinib, verteporfin or combination.

To demonstrate the combination strategy of dual YAP/MEK treatment *in vivo*, we generated a xenograft mouse model through subcutaneously injecting shNf1-SW10 cells into nude mice. We found that the combination of selumetinib and verteporfin significantly inhibited tumor growth compared with selumetinib or verteporfin alone (Figure 7A-C). YAP status and CYR61 expression level *in vivo* xenograft models under treatment of selumetinib and verteporfin were also presented by IHC (Figure 7D). These results showed that the combined targeted YAP and MEK might be a potential strategy for the treatment of NF1 related pNF.

## Discussion

Patients with NF1 usually present with a wide spectrum of manifestations, while the most prevalent pathological feature is neurofibroma 19. Since the breakthrough of novel selumetinib 15, remarkable progress has been made towards NF1 related neurofibroma treatment. Although selumetinib shield light on the NF1 patients, great challenges remain to be addressed: 1) Concerning the genetic nature of lifelong pNF development, continuous treatment may be extended over years 11. 2) The long-term tolerability (or so-called resistance) cannot be ignored, while the individualized administration duration and dosage required to preserve clinical efficacy of optimal MEK inhibition are largely unknown 10. 3) The response rate of selumetinib is now claimed as 68%, which still implies the undesirable effects in about 1/3 of the whole disease population 11. Cautions should be taken to monitor the effects of long-term tolerance and the non-responsive population. Targeted therapy of Neurofibromatosis type 1 (NF1) related plexiform neurofibroma (pNF) aiming at MEK molecule has not demonstrated a convincing result for complete disease inhibition, probably due to other signal pathways crosstalk. To address these concerns, we identified the relationship between Hippo-YAP activation and selumetinib treatment through RNA-sequencing.

Increasing evidence demonstrates that YAP nuclear translocation is involved in cancer initiation and progression 16, 20. The Hippo pathway has been identified as a possible modifier for promoting neurofibroma tumorigenesis 21, and dampening the activity of YAP might be an attractive therapeutic target 14. Additionally, it also has been reported that YAP may be associated with resistance to cancer target therapy in cancer cells harboring NRAS, KRAS, or BRAF mutations 22-25. Kurppa et al. demonstrated that YAP/TEAD/SLUG activation can induce dormancy which facilitate resistance to EGFR/MEK inhibition in NSCLC 23. As for neuroblastoma with hyperactivated RAS activity, YAP might promote the transcriptional activation and expression of E2F and MYC, thereby indirectly mediating trametinib (MEK inhibitor) resistance 26. NF1 plays an inhibitory role in regulating the MAPK/MEK pathway in neurofibroma, and it is also known that there is tight signal cross-talk between the MAPK/MEK and Hippo pathways in some cancers 27, 28. Hippo/YAP and MAPK/MEK signaling pathways share several mechanisms to regulate cellular proliferation and apoptosis. Based on previous studies and the current status of selumetinib, it is reasonable that NF1 related neurofibroma cells may alter the efficacy of selumetinib treatment by regulating the functional state of YAP. However, the mechanism of YAP modulating selumetinib resistance in neurofibroma is currently poorly understood. The Hippo/YAP pathway exerts a critical role in driving neurofibroma development 21. Accordingly, targeting the pathway may introduce a new and promising measure in the current armament 14, 29. Although pharmacologically inhibiting YAP has become a promising goal for tumor therapy, no directly YAP inhibitors have been investigated in clinic. However, it has reported that some drugs could inhibit YAP activity effectively 30. Among them, verteporfin is proved to bind with the conserved TEAD interaction domain in YAP, disrupts YAP-TEAD binding, and induces YAP/TAZ protein degradation, preventing transcriptional transactivation 31,32. Krishanthan Vigneswaran and colleges also demonstrated that verteporfin blocks association between TAZ and TEAD4 and inhibits TAZ-mediated transcriptional activation of TEAD targets in their recent study 33. Therefore, verteporfin was chosen as a promising YAP inhibitor in our study.

Our results showed that selumetinib treatment induced YAP nuclear accumulation, and YAP may play a role in sensitivity to selumetinib* in vitro*. Genetic and/or pharmacologically inhibition of YAP also indicated YAP might enhance the drug-resistant ability of tumor cells. Notably, the CI values also revealed synergistic effects in dual targeting of YAP and MEK signaling. The data provided rational evidence for the development and testing of a YAP inhibitor together with a MEK inhibitor to improve the magnitude and duration of response to MAPK pathway blockade in NF1 related neurofibroma patients. Activated YAP can interact with nuclear transcription factors (mainly TEAD family) to promote transcription of multiple downstream genes 34. Therefore, we demonstrated the synergistic effects of verteporfin and selumetinib for increasing the cessation effects to NF1 related pNF. The selumetinib tolerance can be probably reversed by using verteporfin. Thus, the design and development of combining novel drugs targeting both YAP and MEK be urgently necessary in consideration of a much larger population who can garner benefits from such attempt.

In conclusion, selumetinib treatment promoted the nuclear translocation and transcription activation of YAP in neurofibroma tumor cells. YAP inhibition sensitized neurofibroma tumor cells to selumetinib *in vitro* and *in vivo*. This study provides validated evidence that dual targeting YAP and MEK might be a promising therapeutic strategy for NF1 related pNF.

## Supplementary Material

Supplementary figures and tables.

## References

[1] Gutmann DH, Ferner RE, Listernick RH, Korf BR, Wolters PL, Johnson KJ (2017). Neurofibromatosis type 1. Nat Rev Dis Primers.

[2] Jiang C, McKay RM, Le LQ (2021). Tumorigenesis in neurofibromatosis type 1: role of the microenvironment. Oncogene.

[3] Mo J, Moye SL, McKay RM, Le LQ (2022). Neurofibromin and suppression of tumorigenesis: beyond the GAP. Oncogene.

[4] Mautner VF, Asuagbor FA, Dombi E, Fünsterer C, Kluwe L, Wenzel R (2008). Assessment of benign tumor burden by whole-body MRI in patients with neurofibromatosis 1. Neuro Oncol.

[5] Khu KJ, Midha R (2016). Malignant Peripheral Nerve Sheath Tumors. World Neurosurg.

[6] Boyd KP, Korf BR, Theos A (2009). Neurofibromatosis type 1. J Am Acad Dermatol.

[7] Canavese F, Krajbich JI (2011). Resection of plexiform neurofibromas in children with neurofibromatosis type 1. J Pediatr Orthop.

[8] Gutmann DH, Blakeley JO, Korf BR, Packer RJ (2013). Optimizing biologically targeted clinical trials for neurofibromatosis. Expert Opin Investig Drugs.

[9] Dombi E, Baldwin A, Marcus LJ, Fisher MJ, Weiss B, Kim A (2016). Activity of Selumetinib in Neurofibromatosis Type 1-Related Plexiform Neurofibromas. N Engl J Med.

[10] Gross AM, Dombi E, Widemann BC (2020). Current status of MEK inhibitors in the treatment of plexiform neurofibromas. Childs Nerv Syst.

[11] Gross AM, Wolters PL, Dombi E, Baldwin A, Whitcomb P, Fisher MJ (2020). Selumetinib in Children with Inoperable Plexiform Neurofibromas. N Engl J Med.

[12] Hall A, Choi K, Liu W, Rose J, Zhao C, Yu Y (2019). RUNX represses Pmp22 to drive neurofibromagenesis. Sci Adv.

[13] Wu J, Liu W, Williams JP, Ratner N (2017). EGFR-Stat3 signalling in nerve glial cells modifies neurofibroma initiation. Oncogene.

[14] Liu JL, You YH, Tian ZW, Xiao M, Zheng JW, Wang YA (2021). Increased nuclear translation of YAP might act as a potential therapeutic target for NF1-related plexiform neurofibroma. Int J Med Sci.

[15] Weiss BD, Wolters PL, Plotkin SR, Widemann BC, Tonsgard JH, Blakeley J (2021). NF106: A Neurofibromatosis Clinical Trials Consortium Phase II Trial of the MEK Inhibitor Mirdametinib (PD-0325901) in Adolescents and Adults with NF1-Related Plexiform Neurofibromas. J Clin Oncol.

[16] Dey A, Varelas X, Guan KL (2020). Targeting the Hippo pathway in cancer, fibrosis, wound healing and regenerative medicine. Nat Rev Drug Discov.

[17] Grabinski N, Ewald F, Hofmann BT, Staufer K, Schumacher U, Nashan B (2012). Combined targeting of AKT and mTOR synergistically inhibits proliferation of hepatocellular carcinoma cells. Mol Cancer.

[18] Chou TC (2010). Drug combination studies and their synergy quantification using the Chou-Talalay method. Cancer Res.

[19] Hirbe AC, Gutmann DH (2014). Neurofibromatosis type 1: a multidisciplinary approach to care. Lancet Neurol.

[20] Yu FX, Zhao B, Guan KL (2015). Hippo Pathway in Organ Size Control, Tissue Homeostasis, and Cancer. Cell.

[21] Chen Z, Mo J, Brosseau JP, Shipman T, Wang Y, Liao CP (2019). Spatiotemporal Loss of NF1 in Schwann Cell Lineage Leads to Different Types of Cutaneous Neurofibroma Susceptible to Modification by the Hippo Pathway. Cancer Discov.

[22] Lin L, Sabnis AJ, Chan E, Olivas V, Cade L, Pazarentzos E (2015). The Hippo effector YAP promotes resistance to RAF- and MEK-targeted cancer therapies. Nat Genet.

[23] Kurppa KJ, Liu Y, To C, Zhang T, Fan M, Vajdi A (2020). Treatment-Induced Tumor Dormancy through YAP-Mediated Transcriptional Reprogramming of the Apoptotic Pathway. Cancer Cell.

[24] Shim J, Lee JY, Jonus HC, Arnold A, Schnepp RW, Janssen KM (2020). YAP-Mediated Repression of HRK Regulates Tumor Growth, Therapy Response, and Survival Under Tumor Environmental Stress in Neuroblastoma. Cancer Res.

[25] Sun T, Mao W, Peng H, Wang Q, Jiao L (2021). YAP promotes sorafenib resistance in hepatocellular carcinoma by upregulating survivin. Cell Oncol (Dordr).

[26] Coggins GE, Farrel A, Rathi KS, Hayes CM, Scolaro L, Rokita JL (2019). YAP1 Mediates Resistance to MEK1/2 Inhibition in Neuroblastomas with Hyperactivated RAS Signaling. Cancer Res.

[27] Reddy BV, Irvine KD (2013). Regulation of Hippo signaling by EGFR-MAPK signaling through Ajuba family proteins. Dev Cell.

[28] Feng R, Gong J, Wu L, Wang L, Zhang B, Liang G (2017). MAPK and Hippo signaling pathways crosstalk via the RAF-1/MST-2 interaction in malignant melanoma. Oncol Rep.

[29] Brosseau JP, Liao CP, Le LQ (2020). Translating current basic research into future therapies for neurofibromatosis type 1. Br J Cancer.

[30] Gronich N, Rennert G (2013). Beyond aspirin-cancer prevention with statins, metformin and bisphosphonates. Nat Rev Clin Oncol.

[31] Liu-Chittenden Y, Huang B, Shim JS, Chen Q, Lee SJ, Anders RA (2012). Genetic and pharmacological disruption of the TEAD-YAP complex suppresses the oncogenic activity of YAP. Genes Dev.

[32] Gibault F, Bailly F, Corvaisier M, Coevoet M, Huet G, Melnyk P (2017). Molecular Features of the YAP Inhibitor Verteporfin: Synthesis of Hexasubstituted Dipyrrins as Potential Inhibitors of YAP/TAZ, the Downstream Effectors of the Hippo Pathway. ChemMedChem.

[33] Vigneswaran K, Boyd NH, Oh SY, Lallani S, Boucher A, Neill SG, er al (2021). YAP/TAZ Transcriptional Coactivators Create Therapeutic Vulnerability to Verteporfin in EGFR-mutant Glioblastoma. Clin Cancer Res.

[34] Wu LMN, Deng Y, Wang J, Zhao C, Wang J, Rao R (2018). Programming of Schwann Cells by Lats1/2-TAZ/YAP Signaling Drives Malignant Peripheral Nerve Sheath Tumorigenesis. Cancer Cell.

