# Supplementary material for: Inhibition of YAP Sensitizes the Selumetinib Treatment for Neurofibromatosis Type 1 Related Plexiform Neurofibroma
Source: Int J Med Sci. 2023 Jan 1;20(1):125–35. doi: 10.7150/ijms.78386 (PMC9812799; doi:10.7150/ijms.78386)
Supplement: Supplementary file 1 — Supplementary figures and tables. [file ijmsv20p0125s1.pdf]

Supplementary Materials:

Table S1. The primers sequences used in our study.

Table S2. All the Hippo pathway genes expression level after selumetinib treatment in ipNF95.6 Schwann cells.

Figure S1. *Nf1* deficient mouse Schwann cell model. A lentiviral vector with shRNA was used to knock down *Nf1* in murine Schwann cell line SW10 cells. Transfected efficiency was identified through qRT-PCR(A) and western blot(B). (C) Transfection efficiency was detected using Western blot after YAP-specific siRNA transfection for 48h in neurofibroma Schwann cells and shNf1-SW10 cells.

Figure S2. Sphere sizes were examined in tumor Schwann cells after treating with selumetinib, siYAP or combination (A), also for treating with selumetinib, verteporfin or combination(B). *$p < 0.05$, **$p < 0.01$, ***$p < 0.005$.

Figure S3. Cleaved-caspase3 activities of ipNF95.11bC (A) and ipNF95.6(B) transfected with siYAP were analyzed after selumetinib treatment for 72h.

Figure S4. Flow plots with gating to show how the proportion of apoptotic cells changed after using PI and Annexin V staining.

Figure S5. RNA expression of CTGF/CYR61 in ipNF95.11bC Schwann cells treated with selumetinib, siYAP, selumetinib + siYAP; and also, for selumetinib, verteporfin, combination.

Figure S6. Tumor growth curve of the four different groups. (Vector, selumetinib, verteporfin or combination group)

Table S1. The primers used in our study

Gene symbol	Primer	Sequence (5'-3')
Nf1 (Mouse)	Forward	TGCTGGGCAACCAAAGGAC
	Reverse	TGCGACTAAAGACTGCATTGAAG
Yap1 (Mouse)	Forward	ACCCTCGTTTTGCCATGAAC
	Reverse	TGTGCTGGGATTGATATTCCGTA
Ctgf (Mouse)	Forward	GGCCTCTTCTGCGATTTCG
	Reverse	GCAGCTTGACCCTTCTCGG
Cyr61 (Mouse)	Forward	TAAGGTCTGCGCTAAACAACTC
	Reverse	CAGATCCCTTTCAGAGCGGT
Gapdh (Mouse)	Forward	AGGTCGGTGTGAACGGATTTG
	Reverse	GGGGTCGTTGATGGCAACA
YAP1 (Human)	Forward	TAGCCCTGCGTAGCCAGTTA
	Reverse	TCATGCTTAGTCCACTGTCTGT
CTGF (Human)	Forward	CAGCATGGACGTTCGTCTG
	Reverse	AACCACGGTTTGGTCCTTGG
CYR61 (Human)	Forward	CTCGCCTTAGTCGTCACCC
	Reverse	CGCCGAAGTTGATTCCAG
GAPDH (Human)	Forward	GGAGCGAGATCCCTCCAAAAT
	Reverse	GGCTGTTGTCATACTTCTCATGG

Table S2. Hippo pathway genes expression level in neurofibroma Schwann cells (ipNF95.6). (Vector vs Selumetinib)

Gene_ID	PN956_1	PN956_2	PN956_3	PN956_4	PN_Selu_1	PN_Selu_2	PN_Selu_3	PN_Selu_4
TEAD4	6.981905634	7.032273747	7.082981581	7.050773086	9.567975882	9.53630443	9.539909812	9.43488645
YWHAQ	12.85775194	12.84861033	12.85036709	12.85285659	13.40508251	13.46553656	13.42725436	13.46095917
PPP2R1A	12.06255809	11.98878841	12.0407375	12.04417138	13.38018021	13.34166985	13.35332482	13.2779561
ACTG1	16.52166717	16.4937034	16.48725951	16.51336656	17.3472058	17.33952758	17.33270438	17.27533427
DVL3	11.74687197	11.71246141	11.65649404	11.70240314	12.96795171	12.96088985	12.91899872	12.88467506
AJUBA	9.858331952	9.898696872	9.842985127	9.730112666	12.97529745	13.24763372	13.06458025	12.86164718
FGF1	9.229141102	9.271180903	9.469788274	9.413014106	11.59368815	11.703688	11.61776861	11.52846596
LATS2	9.649745637	9.806954706	9.610943021	9.680293351	11.50424314	11.43167465	11.41774651	11.35956428
YWHAH	11.87561459	11.87304834	11.88315586	11.84636723	12.99668611	13.13943767	13.03416809	12.94728817
TP53BP2	10.13881386	10.18433025	10.14990151	10.2145574	11.52899868	11.70943342	11.50512352	11.62906436
ID2	9.555991091	9.803514103	9.706682092	9.647472056	11.86459043	12.14894092	12.13135531	12.0892081
TGFB3	9.173565941	9.143258462	9.202844526	9.222340962	11.20730453	11.36769505	11.10425323	10.98093111
PPP2R2B	1.951174149	2.449593595	1.551196551	1.932507881	7.645259215	8.235614833	7.88612937	7.790501809
TCF7L1	4.628020187	5.17630274	4.939155111	5.536142577	8.94162368	8.99659921	9.017401551	8.954974649
PPP2CB	10.57724603	10.59977927	10.61050335	10.57539802	11.40439889	11.3950499	11.31133757	11.26439041
SMAD3	11.01696737	10.95541066	10.91904208	10.9708297	11.54927713	11.57994342	11.52551576	11.52201397
WTIP	8.066139925	8.084015332	8.165027909	7.837439458	10.48935413	10.51555045	10.43390536	10.21125923
TEAD3	10.81212491	10.94308051	10.88519873	10.82836242	12.11527166	12.13729142	12.15975496	11.9411017
PPP1CA	10.98648435	10.9746213	10.97268023	11.09018012	11.75397352	11.72098567	11.74070932	11.66383513
TEAD2	8.590756172	8.669120647	8.506473489	8.474382009	9.751039935	9.83670485	9.660260378	9.730622342
SAV1	9.3544322	9.283374131	9.300911527	9.304869806	9.940866221	9.943068144	9.874070677	9.842965423
CTNNA1	13.56623987	13.57463568	13.53789017	13.57021762	14.25131095	14.38738461	14.32915911	14.17109494
LIMD1	9.210453225	9.312181607	9.250249571	9.368421139	10.37880339	10.5833953	10.50511915	10.34623658
RASSF1	9.75683518	9.595244601	9.747205158	9.698487499	11.08755453	11.2671338	11.04861928	10.92957653
LLGL2	1.870480023	1.846995779	2.33707339	2.398588665	5.706485837	5.586518496	5.209005087	5.764321788
CSNK1D	11.37464322	11.33517497	11.25303939	11.36201177	12.24571278	12.26848856	12.21149117	12.08316217
BBC3	7.478589559	7.71991476	7.537904866	7.576814127	9.249251689	9.18910959	8.957721183	9.011843624
YWHAG	12.65162764	12.66552091	12.68736432	12.66167957	13.20001917	13.26575274	13.21589976	13.0936838
AXIN1	9.04601974	9.092464995	9.048452284	9.121443043	10.12635726	10.21302911	10.17226748	9.881135274
FZD5	4.691514157	5.063068409	4.592803382	5.23647616	7.334698733	7.455007062	7.308418993	7.129127564
FZD7	10.63048542	10.6524506	10.44745839	10.54030726	11.86258014	12.05751248	11.87098186	11.6823658
PARD6G	7.061219016	6.872648008	6.969797049	6.917182453	7.781858827	7.909000769	7.874629391	7.722285116
CDH1	4.500139154	3.658442376	3.365195887	3.161822453	7.243772007	7.19973151	7.476726309	7.27956106
FBXW11	10.56707097	10.58102772	10.56543002	10.6076672	11.12097993	11.18884064	11.03652538	11.00891524
TGFB2	10.67618759	10.75223179	10.75983742	10.67553054	12.19172749	11.72067913	12.0465448	12.30297133
FZD4	8.750153855	8.803481116	8.705272969	8.643950348	9.891597085	9.533266149	9.65827034	9.794077891
AXIN2	6.829539137	6.982757986	7.055946385	6.636180338	8.187798602	8.515310195	8.421163553	8.268236434
NF2	10.54432274	10.50195321	10.47249568	10.49335213	10.95278105	10.91933043	10.94045298	10.81575763
APC2	5.368977931	4.661764885	4.704692661	4.830720423	7.392066637	7.287930825	6.920320442	7.175817503
WNT2	-0.536770623	-0.542021682	-0.55345273	-0.540890826	1.168635967	0.439081968	0.728746719	0.695204937
WNT9A	2.216122807	2.84576724	2.136638958	2.196442623	4.761472131	5.223013255	4.602644878	4.78716297

DVL2	9.958856231	9.838030413	9.869794402	9.937591369	10.61330366	10.5295929	10.53487654	10.3880754
YWHAE	12.84870202	12.80024932	12.84235498	12.78172278	13.28703166	13.31560198	13.27827947	13.13385462
PARD6A	3.662741678	3.404714383	3.946228355	4.244303556	6.380023837	5.758800707	5.98082142	5.981141858
BTRC	9.202732179	9.284969491	9.182030096	9.200158769	9.554325485	9.523406144	9.522859298	9.631253854
WNT10B	3.69682374	4.133549467	3.807874721	3.438972459	5.072982131	5.055532193	5.060375908	4.885018157
LLGL1	10.54381468	10.54649701	10.47798865	10.46634534	10.90811194	10.81509507	10.92868007	10.77965875
WNT2B	6.068719788	5.636280367	6.053105093	5.953685464	6.826230012	7.077581406	6.893418132	7.148216083
PATJ	6.511712204	6.227417595	6.324828354	6.067529036	7.329958928	7.164357459	7.460973019	7.614338736
YWHAB	12.55500069	12.59809576	12.56462681	12.51563879	12.87872452	12.78607999	12.81185517	12.75828955
TGFB1	11.75472537	11.73942482	11.72619578	11.7610262	12.08768424	11.92051092	11.96652407	12.02068415
CCND2	4.689040585	5.227759402	5.308580502	5.273484404	6.447808387	6.433387267	6.756220547	6.894177998
WWC1	7.326317833	7.087372299	7.203405932	7.319721911	7.751828434	7.873103093	7.862424999	7.674526704
DLG3	8.307807366	8.245918678	7.952603597	8.030527874	8.737362542	8.889773074	8.874300827	8.733036634
WNT10A	1.34210759	1.320854773	1.274623999	1.325430701	1.457039059	1.513656792	1.482137802	1.433918202
SOX2	1.746520378	1.723343727	1.672997611	1.728332152	1.872221227	1.934382856	1.89975742	1.846882822
BMP4	1.752059287	1.36284457	1.320118343	1.735157078	3.072898027	3.714167456	3.548861016	2.66061384
PPP2R2D	9.285538031	9.366212271	9.383094007	9.354977655	9.696938711	9.761282215	9.656880593	9.517345892
BMPR1A	9.769300971	9.840106309	9.788222167	9.898781682	10.40250147	10.3966287	10.49649927	11.03393298
WNT7A	0.742027693	0.728493135	0.699001407	1.178379074	2.609138879	2.670460514	2.229531262	1.629018406
AMOT	3.286202124	3.109304547	2.706934022	3.26358482	4.133618793	4.550896116	5.210484709	4.356482174
WNT3	6.129016653	6.237957809	6.225309353	6.282574919	6.53629357	6.743719441	6.627606219	6.446130137
SCRIB	10.91012507	10.87868489	10.82779872	10.83857856	11.20819686	11.13403032	11.09950296	10.98247828
LEF1	5.869823866	6.098826391	6.551472622	6.399637409	6.769128359	6.964108746	6.885167585	6.939247092
YWHAZ	13.5234318	13.55186417	13.60877142	13.56061223	13.77806207	13.69888238	13.81877119	13.99230941
DVL1	11.14573539	11.17593616	11.08271091	11.15076975	11.39590877	11.4636786	11.28038434	11.26212851
TGFBR1	9.26476665	9.175682884	9.122444186	9.124339217	9.313654975	9.478172335	9.793758318	10.05386909
FZD9	4.414772393	3.483505447	3.63545168	3.701433167	5.079926214	5.464866554	5.197997936	4.195841627
FZD1	10.04930358	10.1180515	10.08378749	10.17796717	10.49254572	10.2389671	10.37757274	10.20299515
CTNNA3	0.489167526	0.47664149	0.921894295	0.479340415	2.260331894	0.58936524	2.48280017	2.011729636
PPP2R2A	10.13384228	10.13338796	10.11585607	10.00556895	10.21755054	10.15904316	10.22312606	10.2058057
BMP8B	1.738164804	1.715097125	1.664985891	2.030030514	2.182319006	2.524805211	1.8906674	2.66423295
SMAD1	7.307116123	7.539007105	7.279200342	7.411033271	7.485312511	7.640537363	7.671955405	7.895474716
CSNK1E	4.553469456	4.582682847	4.121733042	4.266000609	4.83339246	5.218298961	4.869548436	4.556747613
ITGB2	1.085664236	1.068645539	1.43623799	1.81855741	2.238854589	2.005606166	1.62487442	1.926061291
WNT4	0.771427767	0.257661095	0.233532751	0.260043171	0.848633508	0.886680593	0.86549941	1.830841713
WNT6	1.585623804	1.171668969	1.128210593	1.175968326	1.704954772	1.352459517	1.731069192	2.017921642
PPP2R2C	2.148646682	2.376709713	1.779287266	1.835913349	2.284664342	2.352124408	2.314531031	2.515811655
PARD3	9.24028694	9.217488994	9.081494447	9.254605339	9.27551375	9.463271006	9.351369234	9.250330436
PARD6B	2.826323035	2.386700048	1.79241218	2.805735309	2.771181651	2.840474098	3.177931521	3.418515067
FZD3	2.81931916	2.594441803	2.062993872	2.122356036	2.538494657	2.839068644	3.494302376	2.938354184
CCND3	10.48364827	10.53008688	10.4636495	10.52287945	10.54514121	10.56438954	10.54821506	10.49337351
GDF7	-0.402983299	0.069040257	-0.419999718	-0.407189506	-0.374009771	-0.359767797	0.14132129	1.403177846
TCF7L2	8.716414486	8.733662572	8.666005987	8.694259061	8.696702451	8.743608184	8.685079082	8.852175243
PRKCZ	4.512136405	5.004173335	4.593860327	5.010755543	4.676150899	5.146199083	5.050508763	4.929197422

TCF7	6.183113424	6.417096392	6.177491427	5.855470703	5.785871864	6.674517789	6.541229788	6.396234572
BMP8A	2.94640527	2.137707579	1.094565943	1.536729156	1.656944537	2.053607081	2.307909067	2.49803013
AFP	3.474601012	3.181637673	3.806350876	4.047267123	4.45575288	3.27398262	3.388908393	4.021340283
WNT7B	3.090725492	2.901385976	3.283507597	3.975891634	3.389888656	4.150379465	2.480267211	3.62045195
FZD6	9.188721627	9.286909428	9.283764919	9.149591945	8.937138661	9.146627701	9.179570797	9.68198833
BMP7	1.364757417	1.34422166	1.299550673	2.025411279	1.475810502	1.53051686	1.500062018	1.453470001
CTNNB1	12.17525908	12.24083673	12.20034265	12.17510002	12.23574322	12.18474807	12.18064917	12.17871702
SMAD4	10.33498882	10.27915554	10.31828087	10.32918865	10.28421709	10.28896913	10.14834819	10.49058972
TP73	0.579215698	0.566380369	0.538392591	1.036580153	0.648261094	0.682028613	0.663251442	0.634423164
CRB2	0.139369898	1.045340639	0.105911254	1.048851672	0.195700445	1.194236708	0.207884051	0.184438376
SMAD2	10.38239525	10.42571543	10.3940972	10.3599346	10.12174801	10.29247123	10.34672939	10.52844537
PPP2R1B	8.843044853	9.062136677	8.979478265	9.046497182	8.749538803	8.76395146	8.66158889	9.221499801
YAP1	12.24360454	12.2922483	12.27445717	12.22366473	12.06370435	12.10345963	12.1233864	12.40961747
PRKCI	9.600403716	9.719413393	9.615613826	9.706789069	9.205755251	9.268545217	9.320342946	9.993967531
BMP5	0.479377242	1.29355801	0.900479838	1.590557554	0.538629933	0.56765477	1.03825621	0.526744028
NKD1	2.120029965	2.097606571	2.287688164	1.820273772	2.242048128	1.225395532	1.97692503	1.161234326
BMP2	6.244235192	6.447534364	6.867156784	6.487395352	5.901697301	5.86994925	6.57059151	6.30206087
GSK3B	10.67771056	10.80358899	10.81766614	10.71873726	10.426099	10.41469551	10.48728597	10.90626672
FZD2	11.67203533	11.63614922	11.6797063	11.66620496	11.69158774	11.64735459	11.52104249	11.44897647
SERPINE1	16.14577729	16.12406848	16.11198941	16.12058673	16.16698415	16.06182456	16.00837293	15.83980998
STK3	8.933143426	9.115060697	9.033008869	9.061577497	8.659258148	8.828198734	8.917803506	8.934792331
PPP1CC	10.77405892	10.74013709	10.7910354	10.77604714	10.49126121	10.55785999	10.58950994	10.79177879
PPP2CA	11.5403876	11.55621605	11.58933454	11.56981974	11.51325653	11.48531964	11.43512349	11.53194071
BMPR2	11.53809992	11.59197597	11.58623994	11.55927545	10.49188554	10.55731172	10.94616785	11.66539916
DLG1	10.85675402	10.86955932	10.79234829	10.82498069	10.43714047	10.32924424	10.55569809	10.7512838
FZD8	7.921109317	8.288246992	8.039301302	8.138885788	7.436475364	7.83999293	7.495763936	7.390289007
TEAD1	12.22454275	12.27880487	12.28693667	12.23871858	11.3973516	11.31756326	11.6103183	12.09492822
CRB1	3.052602688	4.032268954	3.498108733	3.136827954	1.807603097	1.539983475	2.09521499	0.598787775
SMAD7	10.37938264	10.38272347	10.38127806	10.38307697	10.31552116	10.14898403	10.20579748	10.04537926
WNT11	2.978690575	2.707826798	3.289130748	3.168346657	1.228942511	1.948744749	1.919837147	2.124753978
GLI2	10.644646	10.57105586	10.65893815	10.63012577	10.35136543	10.1589954	10.45086755	10.22594066
AMH	7.482852043	7.508484364	7.478053704	7.333187619	7.082262648	6.599838733	6.369656987	6.620056953
DLG2	4.853055959	5.277794101	4.619099252	4.784460255	3.800266169	3.764287796	4.122170472	3.540901239
WNT5B	9.877519447	9.920068784	9.705461368	9.833789149	9.413166111	9.492592941	9.350322147	9.193078034
RASSF6	1.477826847	1.097346148	1.062984868	1.463782448	0.724363651	0.758601316	0.739559372	0.710340782
LATS1	10.11583413	10.15679837	10.07088116	9.99878595	8.715936413	8.767539315	9.101548831	9.590766411
WNT16	2.948079312	3.476022821	2.770731966	2.594928673	0.870905426	1.316800849	1.616543695	0.855475095
BMPR1B	6.389943601	6.849619991	6.440076604	6.579123135	3.586541278	4.311926872	4.718814497	5.093357447
DLG4	9.571320213	9.442503647	9.494953388	9.37208505	9.006250632	9.022738521	8.95841269	8.773814233
APC	10.49453561	10.56017735	10.63216631	10.51365472	9.333481119	9.781877643	9.831840449	9.880220903
MOB1A	11.41841471	11.44262713	11.45184541	11.38005998	10.61976777	10.63189044	10.70124894	11.05599112
GDF5	7.200507329	7.130691306	7.189600862	7.57840069	4.8521602	5.901656745	5.272568203	5.50335005
PPP1CB	11.66446966	11.7606883	11.76633825	11.73178421	10.86063712	10.95060975	10.9963217	11.29950633
MOB1B	10.72251578	10.89670434	10.84768796	10.74016966	8.119735356	8.282930757	8.690814029	9.228632761

**MYC**	**9.629393723**	**9.547401901**	**9.532812911**	**9.453168721**	**8.970119718**	**8.806001107**	**8.812922708**	**8.898644444**
**FRMD6**	**12.54276055**	**12.49572785**	**12.56458242**	**12.55263445**	**11.46893488**	**11.56449386**	**11.59745395**	**11.86780937**
**GDF6**	**6.450564139**	**6.284341437**	**6.531298965**	**6.409690664**	**4.590630713**	**5.079400741**	**4.626749102**	**4.489165548**
**ID1**	**9.765723617**	**9.748536608**	**9.760185709**	**9.822154234**	**8.165712343**	**7.720373189**	**8.241515789**	**7.981388251**
**AREG**	**7.417041854**	**7.360758589**	**7.275078797**	**6.952424826**	**2.952056967**	**2.822106692**	**2.553932534**	**1.93253366**
**SNAI2**	**11.47373912**	**11.5639455**	**11.57104762**	**11.56121721**	**9.501993776**	**9.062534598**	**9.560805817**	**9.256226096**
**WWTR1**	**11.4267628**	**11.45535591**	**11.5196742**	**11.48576562**	**9.949166989**	**9.899238139**	**10.01356621**	**10.21993761**
**BIRC2**	**11.25826621**	**11.32273433**	**11.3402567**	**11.28705236**	**9.506771709**	**9.606222473**	**9.761573351**	**9.66651622**
**CCND1**	**13.74924971**	**13.81817447**	**13.79358813**	**13.76025367**	**11.79881357**	**11.53746384**	**11.65878002**	**11.47217851**
**BIRC5**	**10.43432438**	**10.30962896**	**10.36767274**	**10.45902971**	**4.283832555**	**4.185454318**	**4.139519865**	**3.631270633**
**BMP6**	**11.51492989**	**11.53966369**	**11.48916529**	**11.4956584**	**5.998893682**	**6.031531201**	**6.395564455**	**5.990012349**
**WNT5A**	**14.47564753**	**14.52563355**	**14.54180795**	**14.49829346**	**11.65579275**	**11.5947772**	**11.79148905**	**11.69838489**
**TGFBR2**	**12.60528834**	**12.6615786**	**12.63319857**	**12.60021485**	**10.80613474**	**10.79501946**	**10.76150718**	**10.7300378**

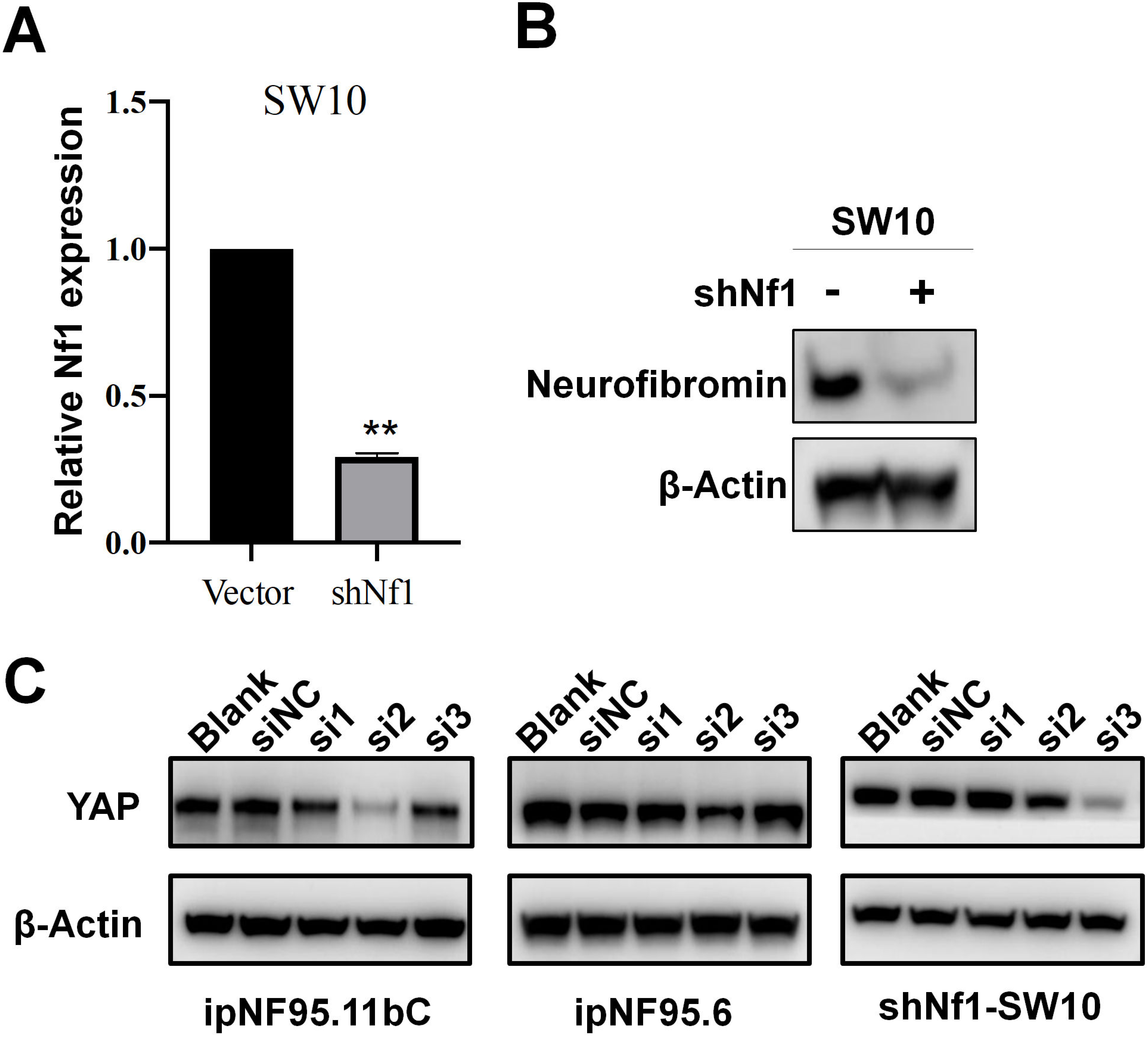
A
SW10
Relative Nf1 expression
1.5
1.0
0.5
0.0
**
Vector
shNf1
B
SW10
shNf1 - +
Neurofibromin
β-Actin
C
Blank siNC si1 si2 si3
YAP
β-Actin
ipNF95.11bC
ipNF95.6
shNf1-SW10

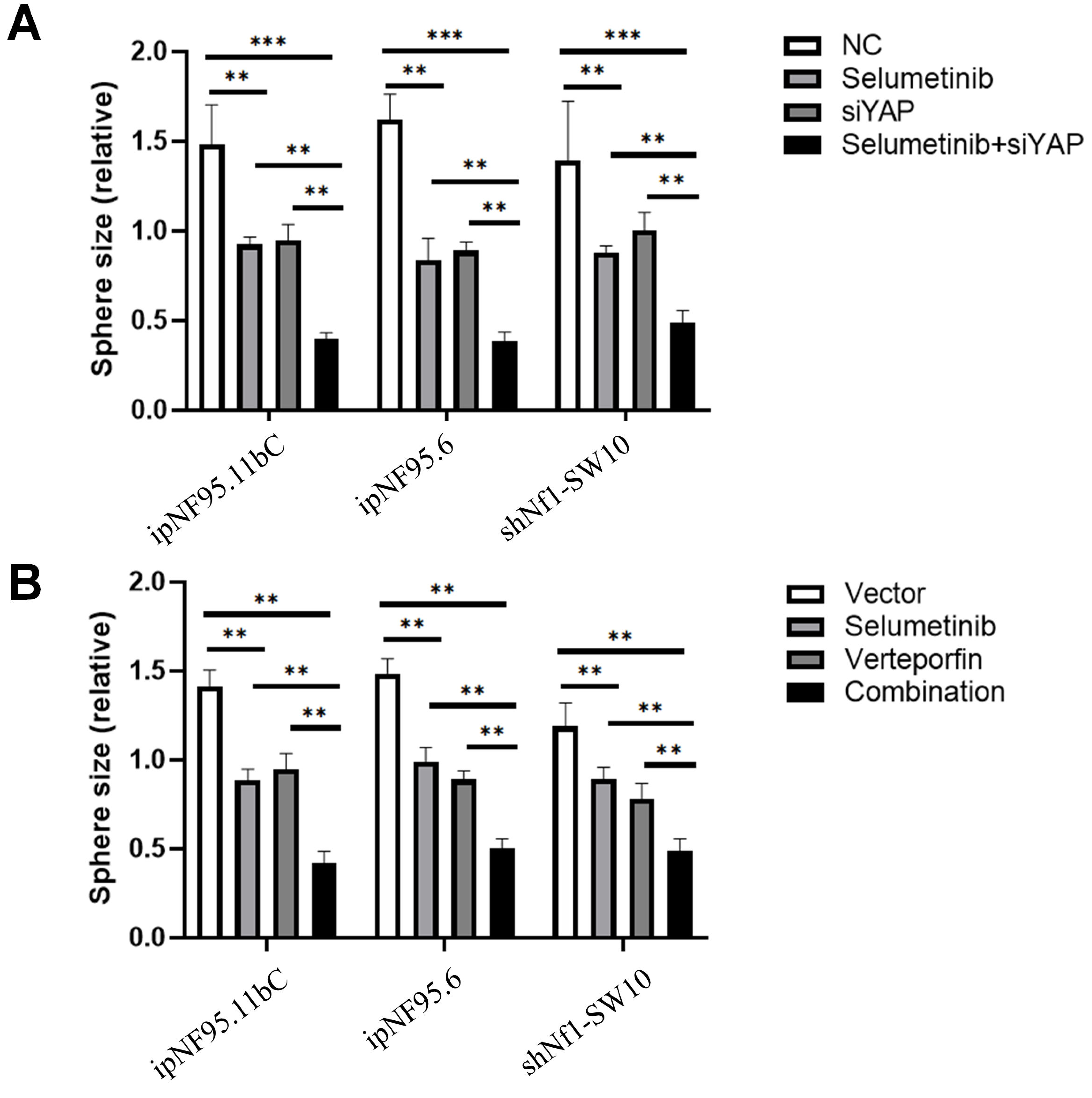
A
NC
Selumetinib
siYAP
Selumetinib+siYAP
Sphere size (relative)
2.0
1.5
1.0
0.5
0.0
***
**
ipNF95.11bC
ipNF95.6
shNf1-SW10
B
Vector
Selumetinib
Verteporfin
Combination

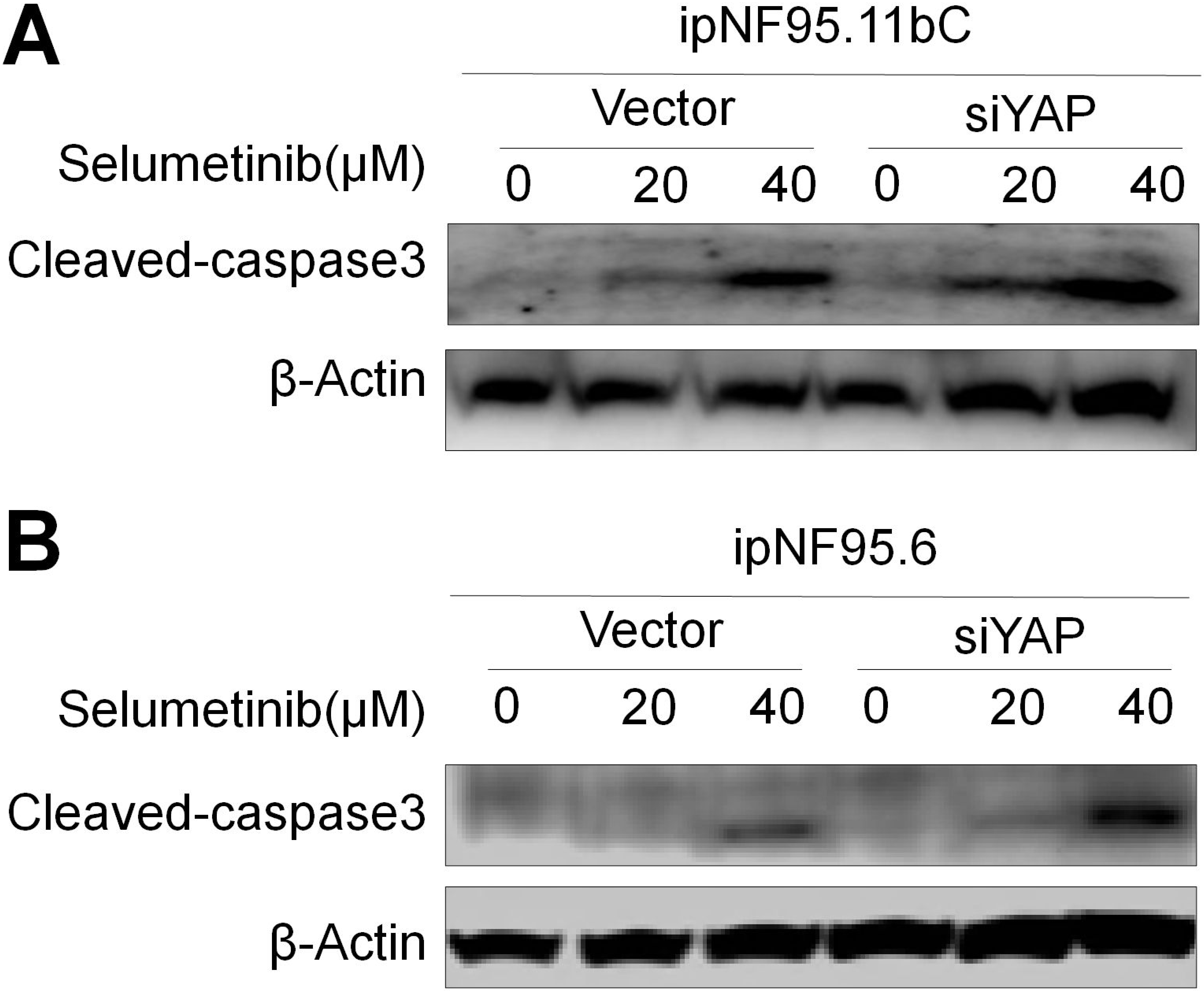
A
ipNF95.11bC
Vector
siYAP
Selumetinib(μM)
0
20
40
0
20
40
Cleaved-caspase3
β-Actin
B
ipNF95.6
Vector
siYAP
Selumetinib(μM)
0
20
40
0
20
40
Cleaved-caspase3
β-Actin

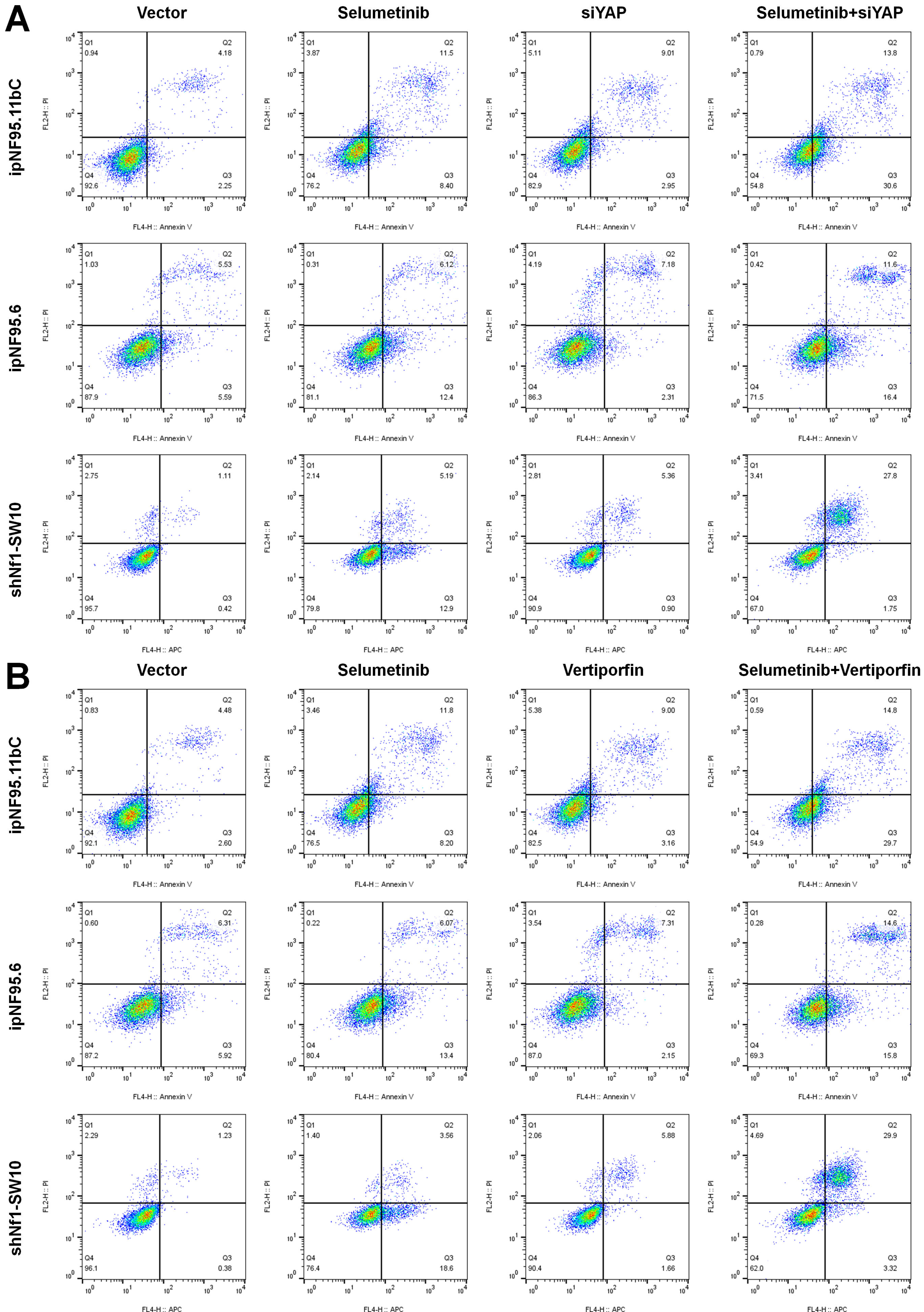
A
Vector
Selumetinib
siYAP
Selumetinib+siYAP
ipNF95.11bC
Q1 0.94 Q2 4.18 Q4 92.6 Q3 2.25
Q1 3.87 Q2 11.5 Q4 76.2 Q3 8.40
Q1 5.11 Q2 9.01 Q4 82.9 Q3 2.95
Q1 0.79 Q2 13.8 Q4 54.8 Q3 30.6
ipNF95.6
Q1 1.03 Q2 5.53 Q4 87.9 Q3 5.59
Q1 0.31 Q2 6.12 Q4 81.1 Q3 12.4
Q1 4.19 Q2 7.18 Q4 86.3 Q3 2.31
Q1 0.42 Q2 11.6 Q4 71.5 Q3 16.4
shNf1-SW10
Q1 2.75 Q2 1.11 Q4 95.7 Q3 0.42
Q1 2.14 Q2 5.19 Q4 79.8 Q3 12.9
Q1 2.81 Q2 5.36 Q4 90.9 Q3 0.90
Q1 3.41 Q2 27.8 Q4 67.0 Q3 1.75
FL2-H :: PI
FL4-H :: Annexin V
FL4-H :: APC
B
Vector
Selumetinib
Vertiporfin
Selumetinib+Vertiporfin
ipNF95.11bC
Q1 0.83 Q2 4.48 Q4 92.1 Q3 2.60
Q1 3.46 Q2 11.8 Q4 76.5 Q3 8.20
Q1 5.38 Q2 9.00 Q4 82.5 Q3 3.16
Q1 0.59 Q2 14.8 Q4 54.9 Q3 29.7
ipNF95.6
Q1 0.60 Q2 6.31 Q4 87.2 Q3 5.92
Q1 0.22 Q2 6.07 Q4 80.4 Q3 13.4
Q1 3.54 Q2 7.31 Q4 87.0 Q3 2.15
Q1 0.28 Q2 14.6 Q4 69.3 Q3 15.8
shNf1-SW10
Q1 2.29 Q2 1.23 Q4 96.1 Q3 0.38
Q1 1.40 Q2 3.56 Q4 76.4 Q3 18.6
Q1 2.06 Q2 5.88 Q4 90.4 Q3 1.66
Q1 4.69 Q2 29.9 Q4 62.0 Q3 3.32
FL2-H :: PI
FL4-H :: Annexin V
FL4-H :: APC

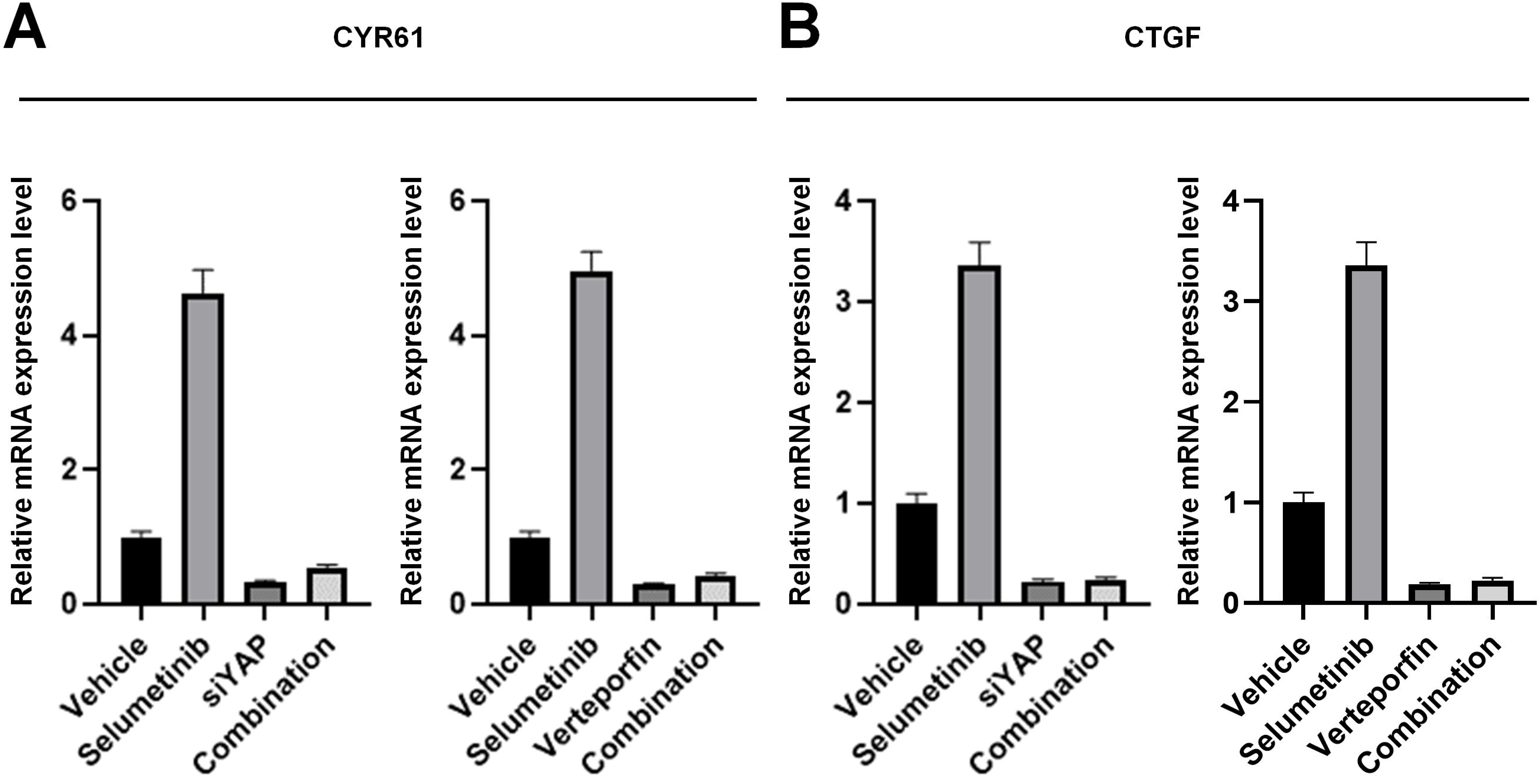
A
CYR61
B
CTGF
Relative mRNA expression level
Vehicle
Selumetinib
siYAP
Combination
Verteporfin

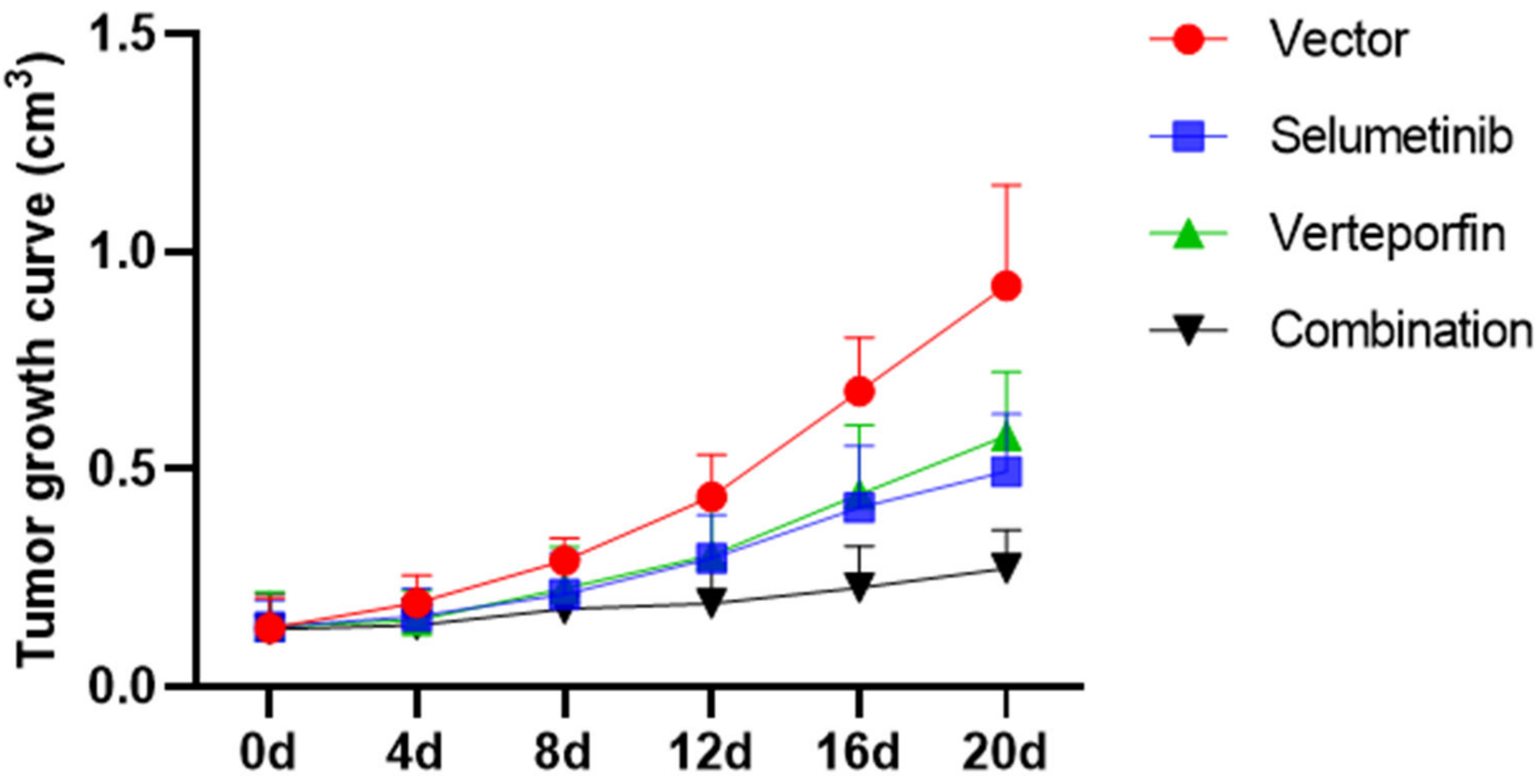

Tumor growth curve (cm^3)
1.5
1.0
0.5
0.0
0d
4d
8d
12d
16d
20d
Vector
Selumetinib
Verteporfin
Combination
